# Prevalence, knowledge, attitudes, and practices concerning self-medication with over-the-counter drugs among university students in Jordan: A cross-sectional study

**DOI:** 10.1371/journal.pone.0339915

**Published:** 2026-01-28

**Authors:** Abdallah Y. Naser

**Affiliations:** Department of Applied Pharmaceutical Sciences and Clinical Pharmacy, Faculty of Pharmacy, Isra University, Amman, Jordan; The University of Jordan School of Pharmacy, JORDAN

## Abstract

**Background:**

The World Health Organization considers self-medication (SM) a critical global concern. SM has significant risks, including incorrect drug use, adverse reactions, misdiagnosis, and increased antibiotic resistance. The prevalence of SM is particularly high in developing countries, where students can purchase medications without medical supervision. This study investigates the prevalence of SM practices using over-the-counter (OTC) drugs and their associated predictors. Additionally, this study explores the knowledge, attitudes, and practices of university students in Jordan regarding SM.

**Method:**

A cross-sectional online survey study was conducted among university students in Jordan from March to September 2024. The questionnaire was distributed among university students through online social media platforms (Facebook, WhatsApp, and Instagram). To identify predictors of SM practice among university students, binary and multiple logistic regression analyses were implemented. A Chi-squared test was used to estimate the risk of SM among students.

**Results:**

Of 1,269 university students participating, 45.3% reported practicing SM. Knowledge about OTC drug safety was generally high, as most students identified the risks of combining OTC and prescription medications (83.5%) and the importance of avoiding expired drugs (95.5%). Medical students demonstrated significantly better knowledge of diseases manageable by OTC drugs and the likelihood of side effects (p < 0.001). Attitudes varied; the majority of students (71%) agreed that a doctor should be consulted before using OTC medications, pharmacists were the primary source of advice (51.2%), and antipyretics were the most commonly used medications (46.9%). Multiple logistic regression provided evidence that fifth-year students were significantly more likely to practice SM (adjusted odds ratio (aOR) = 2.38, p = 0.021). No other demographic variable was statistically associated with this practice.

**Conclusion:**

Fifth-year students exhibited a significantly higher estimated risk of practicing SM compared to their peers in other academic years. This highlights the need for targeted educational interventions to promote safe SM practices. These findings highlight the need for targeted educational interventions. However, the use of self-reported data is a limitation that may introduce recall or reporting bias.

## Introduction

SM is defined as the practice of taking non-prescribed medicine to address self-diagnosed health issues [1]. OTC drugs are medications that can be purchased without a prescription and are generally considered safe and appropriate for use without the need for healthcare professional oversight [2]. It is important to distinguish between using OTC medications for self-treatment and obtaining prescription drugs without a doctor’s supervision [3]. The World Health Organization emphasized that, despite its potential benefits, SM also carries risks if practiced improperly [4].

Worldwide, the prevalence of SM is high [5]. It also varies significantly, from 11.2% to 93.7%, depending on the country and population studied [5]. In particular, SM has become increasingly widespread among university students worldwide. This behavior is influenced by multiple factors, including the ease of obtaining OTC medications, the accessibility of pharmacies, financial constraints, particularly among university students with limited budgets, the growing popularity of social media, which significantly impacts SM behavior by facilitating access to information about medications, the use of information from leaflets, and a desire to save time and costs associated with seeking professional medical care [6].

While SM may offer temporary relief, it poses significant risks, such as improper drug use, adverse reactions, misdiagnosis, and increased concerns about antibiotic resistance. It can also lead to an increase in the use of illegal drugs [7]. Previous literature has highlighted that the practice of SM tends to be higher in developing countries [7], where students can purchase medications without medical supervision [8]. A limited number of studies have examined SM in Jordan, specifically among university students. One previous study found that 21.0% of patients with ophthalmic conditions practice SM [9]. Another study in Jordan reported that 56.9% of the general public practiced SM and used medication(s) not prescribed by a healthcare specialist [10]. The most reported indications for SM included headache (86.9%), flu (76.4%), and fever (69.6%) [11]. In 2018, Alsous *et al.* found that 86.7% of pharmacy students practiced SM [12]. Despite several studies addressing SM in Jordan, important gaps remain. Previous research has not evaluated the knowledge, attitudes and practices model among university students. Additionally, the existing literature has did not compare medical and non-medical students. Furthermore, earlier studies were primarily descriptive and did not assess predictors of SM behavior. With the rapid expansion of OTC drug availability and growing reliance on online health information as a valuable source for practicing SM, updated evidence is needed to understand current knowledge levels, perceptions, and actual SM practices among Jordanian university students.

Therefore, the present study seeks to investigate the prevalence of SM using OTC drugs and their associated predictors. This study also explores the knowledge, attitudes, and practices among university students in Jordan regarding SM.

## Materials and methods

### Study design

This cross-sectional online survey study was conducted among university students in Jordan from March to September 2024.

### Study population

The study population comprised university students in Jordan. The inclusion criteria were that the participants should be male or female students currently studying in public or private Jordanian universities. This study targeted all university students, with no restrictions on the field of study or year of study. Participants who did not provide their consent for participation were excluded from the study.

### Participant recruitment

This research used convenience sampling to recruit the study participants. To ensure that university students from different socioeconomic backgrounds were included, the online questionnaire link was disseminated via social media platforms (Facebook, WhatsApp, and Instagram). The questionnaire link and inclusion criteria were included in the first paragraph of the questionnaire. If the inclusion criteria were fulfilled, university students were invited to participate. While this approach enabled the efficient collection of a large sample, convenience sampling may introduce selection bias, as students who are less active on social media or less engaged online may have been underrepresented.

### Questionnaire

The questionnaire used in the present study was derived from one previously developed by Tesfamariam et al. [13]. It was divided into four sections, assessing the demographic characteristics, knowledge of OTC SM, attitude toward OTC SM, and associated practices of university students. The questionnaire, which consisted of ten items (five yes/no format questions and five multiple-choice questions), was used to assess the respondents’ general knowledge of OTC medications concerning indications, contraindications, adverse effects, and proper treatment usage. The section on attitudes employed a five-point Likert scale (ranging from one for ‘strongly disagree’ to five for ‘strongly agree’). It included 11 items that evaluated the perception of safety, efficacy, accessibility, and usage of OTC medications. The higher the score, the more positive the attitude towards SM. The final evaluation of practice entailed the administration of a series of six multiple-choice questions that evaluated the healthcare professional consulted before the practice of OTC SM, the reasons for OTC SM, the categories of medications used for OTC SM, the frequency of adverse effects due to OTC medications, and whether the individual had ever taken OTC medications above the recommended dosage.

### Instrument validity and reliability

The content validity of the questionnaire was confirmed by an expert panel of two clinical pharmacists. The questionnaire was translated from English to Arabic using forward-backward translation. Face validity was conducted through the distribution of the questionnaire to 40 participants in a pilot study to assess its clarity. The expert pilot study showed that no modifications were required. Kaiser-Meyer-Olkin (KMO) and Bartlett’s Test of Sphericity were employed to evaluate factor analysis.

### Ethical approval

The Institutional Review Board (IRB) at Isra University, Amman, Jordan, approved the study: IRB No. SREC/22/12/60. The World Medical Association Declaration of Helsinki was adhered to during this research. All participants in this study provided written informed consent.

### Sample size

According to the latest available statistics released by the Ministry of Higher Education and Scientific Research, the total number of registered university students in Jordan is 254,078 [14]. At a 95% confidence interval, a standard deviation of 0.5, and a margin of error of 5%, the minimum required sample size was 383 students.

### Statistical analysis

The data for this research were analyzed using the Statistical Package for the Social Sciences software, version 29. Frequencies and percentages were employed to represent categorical data. A Chi-squared test was conducted to identify factors associated with SM. The mean SM attitude score was used to define a positive attitude towards SM (29.6), and participants with a mean knowledge score of 14.8 were classified as having good knowledge. To identify predictors of SM practice among university students, binary and multiple logistic regression analyses were implemented. The odds ratio was illustrated with its corresponding 95% confidence interval. An exploratory factor analysis was performed to assess the underlying structure of the knowledge and attitude items. Sampling adequacy was evaluated by KMO statistics and Barlett’s Test of Sphericity. Principal component analysis with direct oblimin rotation was conducted. Factors were retained based on multiple criteria: eigenvalues greater than 1, factor interpretability, and inspection of the scree plot. Items with a factor loading of 0.45 were considered meaningful contributors. Reliability for each factor was assessed using Cronbach’s alpha. A p-value of less than 0.05 was designated as the significance level.

## Results

### Factor analysis and reliability

**Fig 1:**
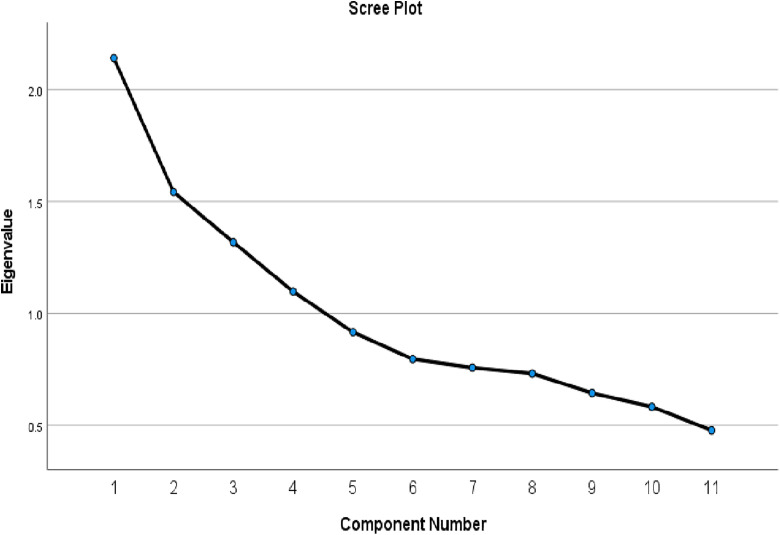
Scree plot for PCA.

To assess the construct validity of the attitude scale, which consists of 11 five-point Likert scale items, exploratory factor analysis using principal component analysis was conducted. Sampling adequacy was confirmed by a borderline-acceptable KMO value of 0.633, and Bartlett’s test of sphericity was significant (X^2^ = 1349.69, p < 0001), indicating suitability for factor analysis. Four factors were extracted with eigenvalues greater than 1, explaining a total of 55.4% of the variance, as shown in the scree plot in Fig 1.

Factor 1 included five items with strong loadings (>0.59) and demonstrated acceptable internal reliability (Cronbach’s alpha = 0.40). Factor 2 contained four items with good loading (>0.45) and good internal reliability (Cronbach’s alpha = 0.57). However, Factors 3 and 4 each contained a single item with a loading factor ≥ 0.45, indicating limited internal structure. Therefore, internal consistency could not be assessed. Despite this, both items were retained because they represent unique conceptual constructs within the survey, Table 1 [15].

**Table 1:** Load factor values of the items of the attitude scale.

Attitude item	Component			
	1	2	3	4
Beyond a month of opening, liquid medications can be used.	0.651	0.105	0.386	−0.337
Temperature, moisture, and direct sunshine do not affect over-the-counter medications.	0.644	−0.164	0.088	0.326
When pregnant, any over-the-counter medications can be used.	0.617	−0.001	−0.179	0.343
Beyond a month of opening, eye/ear drops can be used.	0.595	0.000	0.447	−0.408
When taken on an empty stomach, pain relievers do not result in gastritis.	0.502	−0.123	−0.014	0.450
When taken as an overdose, paracetamol is a potent toxin.	−0.177	0.609	0.041	0.202
Over-the-counter medications can change how another medication works.	0.006	0.572	0.051	0.366
Although over-the-counter medications are safe, you should consult a doctor before using them.	−0.189	0.543	0.431	−0.075
Self-medication with over-the-counter medications is safe.	0.304	0.506	−0.401	−0.324
Over-the-counter medications are more affordable and practical.	0.361	0.475	−0.402	−0.047
I should avoid taking over-the-counter medications when I am sick.	−0.156	0.120	0.646	0.323

### University students’ demographic characteristics

Table 2 presents the demographic characteristics of the 1,269 university students who participated in this study. More than half (60.3%) were females. Two-fifths (41.4%) of the students were in their first year of study. The monthly income for 46.5% of the students was less than JOD 500. More than half of the participants (57.9%) were studying non-medical fields (engineering, computer sciences and information technology, business administration, arts and humanities, sciences, law, agriculture, tourism, and sport sciences). The vast majority of the students (90.3%) were unemployed. Only 8.7% of the students reported a history of chronic disease. Nearly half (45.3%) of the students reported practicing SM.

**Table 2:** Participants’ demographic characteristics (n = 1,269).

Variable	Frequency	Percentage
**Gender**		
Male	504	39.7%
Female	765	60.3%
**Year of study**		
First year	526	41.4%
Second year	360	28.4%
Third year	186	14.7%
Fourth year	114	9.0%
Fifth year	36	2.8%
Sixth year	18	1.4%
Postgraduate	29	2.3%
**Monthly income category**		
Less than JOD 500	590	46.5%
JOD 500–1000	422	33.3%
JOD 1001–1500	136	10.7%
JOD 1501 or more	121	9.5%
**Field of study**		
Medical field (medicine and surgery, dentistry, pharmacy or Doctor of Pharmacy, nursing, allied health sciences, and clinical nutrition and dietetics)	534	42.1%
Non-medical field (engineering, computer sciences and information technology, business administration, arts and humanities, sciences, law, agriculture, tourism, and sport sciences)	735	57.9%
**Employment status**		
Unemployed	1,146	90.3%
Employed	123	9.7%
**Do you have any history of chronic disease?**		
Yes	111	8.7%
No	1,158	91.3%
**Are you currently using or have you used medications not prescribed by a healthcare specialist in the previous year?**		
Yes	575	45.3%
No	694	54.7%

### Knowledge related to SM

Table 3 presents the university students’ responses to the knowledge items. The mean knowledge score was 14.8 (SD: 2.6) out of 20. The knowledge score reflected a moderately high knowledge level (comprising 74.0% of the maximum attainable knowledge score). Three-fifths (60.4%) of the students showed good understanding of SM. The majority were cautious about OTC medications, as 83.5% confirmed that the combination of OTC and prescribed drugs could be risky at times. A higher proportion, 95.5%, reported that OTC medications should not be used after the expiration date, while 81.4% disagreed with the statement that all OTC medications were safe and effective. Accordingly, 93% of the respondents were aware of the caution that should be exercised during pregnancy, 74.5% during lactation, and 70% for both children and the elderly. More importantly, in the event of suspected side effects, 85.8% would report to a doctor or pharmacist, indicating a strong inclination toward professional advice. Students’ SM knowledge concerning the items “diseases can be managed using OTC medications” and “the probability of side effects while using OTC drugs” differed significantly based on their field of study (medical field students versus non-medical field students) (p < 0.001).

**Table 3:** Knowledge related to SM (n = 1,269).

Variable	Overall (n = 1,269)		Medical students (n = 534)		Non-medical students (n = 735)		P-value
	Frequency	Percentage	Frequency	Percentage	Frequency	Percentage	
**A. General medication knowledge**							
Is a doctor’s prescription the only way to use medications? (Yes)	661	52.1%	284	53.2%	377	51.3%	0.531
Are all over-the-counter medications safe and efficient? (No)	1033	81.4%	439	82.2%	594	80.8%	0.559
Are over-the-counter medications permitted for self-care? (No)	431	34.0%	178	33.3%	253	34.4%	0.719
Are all over-the-counter medications safe to take with prescription medications? (No)	1060	83.5%	459	86.0%	601	81.8%	0.055
Can you use over-the-counter medications beyond their expiration date? (No)	1212	95.5%	507	94.9%	705	95.9%	0.414
**B. Diseases can be managed using over-the-counter medications:**							
Hereditary diseases	26	2.0%	9	1.7%	17	2.3%	<0.001
Minor illnesses and injuries§	1006	79.3%	456	85.4%	550	74.8%	
Don’t know	237	18.7%	69	12.9%	168	22.9%	
**C. Which of the following are classified as over-the-counter drugs? (multiple answer question)**							
Antipyretics	953	75.1%	394	73.8%	559	76.1%	0.953
Anti-cold	666	52.5%	287	53.7%	379	51.6%	0.666
Analgesics	614	48.4%	279	52.2%	335	45.6%	0.614
Anti-microbials	373	29.4%	153	28.7%	220	29.9%	0.373
**D. The probability of side effects while using over-the-counter drugs is:**							
Sometimes	759	59.8%	333	62.4%	426	58.0%	<0.001
Mostly	203	16.0%	97	18.2%	106	14.4%	
Never	123	9.7%	56	10.5%	67	9.1%	
Don’t know	184	14.5%	48	9.0%	136	18.5%	
**E. In which of the following situations, when using over-the-counter drugs, should caution be taken? (multiple answer question)**							
Pregnancy	1180	93.0%	493	92.3%	687	93.5%	0.118
Lactation	946	74.5%	416	77.9%	530	72.1%	0.946
Elderly	890	70.1%	386	72.3%	504	68.6%	0.890
Children	892	70.3%	377	70.6%	515	70.1%	0.892
Adolescents/middle-aged adults	528	41.6%	216	40.4%	312	42.4%	0.528
**F. If one observes any possible adverse effects, one should: (multiple answer questions)**							
Report it to a doctor or pharmacist	1089	85.8%	451	84.5%	638	86.8%	0.108
Stop using the drug immediately	960	75.7%	412	77.2%	548	74.6%	0.960
Lower the dose until the side effect(s) stop	81	6.4%	44	8.2%	37	5.0%	0.810
Continue taking the drug	18	1.4%	8	1.5%	10	1.4%	0.180

§ Headaches or mild migraines, common cold and flu, cough, fever, nasal congestion or runny nose, heartburn, diarrhea or constipation, menstrual cramps, or pain.

### Attitudes toward SM practice

Table 4 presents the attitudes of university students toward SM practices. The mean attitude score was 29.6 (SD: 4.4) out of 55. The attitude score reflected a moderate attitude level (comprising 53.8% of the maximum attainable attitude score). Just over half (52.6%) of the students showed a positive attitude towards SM. Agreement on statements that examined attitudes toward SM practices was estimated by summing “Agree” and “Strongly Agree” and ranged from 6.3% to 71%. The statement “Although over-the-counter medications are safe, you should consult a doctor before using them” had the highest agreement, with 71%, thus strongly supporting the preference to consult a doctor even when OTC drugs were perceived as safe. The lowest score, 6.3%, was a response to the statement, “When pregnant, any over-the-counter medications can be used,” meaning that the great majority of the participating students were aware that OTC drugs are not universally safe during pregnancy.

**Table 4:** Attitudes toward SM (n = 1,269).

Attitude statement	Strongly disagree	Disagree	Neutral	Agree	Strongly agree
Self-medication with over-the-counter medications is safe.	15.4%	18.7%	45.2%	19.7%	0.9%
Over-the-counter medications are more affordable and practical.	17.1%	36.8%	27.5%	16.4%	2.2%
When taken as an overdose, paracetamol is a potent toxin.	3.2%	6.9%	39.8%	38.5%	11.7%
Over-the-counter medications can change how another medication works.	7.1%	13.6%	25.8%	43.0%	10.5%
When pregnant, any over-the-counter medications can be used.	52.9%	29.6%	11.2%	4.5%	1.8%
When taken on an empty stomach, pain relievers do not result in gastritis.	31.8%	36.7%	16.5%	12.2%	2.8%
Temperature, moisture, and direct sunshine do not affect over-the-counter medications.	46.3%	32.9%	13.6%	6.3%	0.9%
Beyond a month of opening, liquid medications can be used.	19.5%	30.8%	31.9%	16.1%	1.7%
Beyond a month of opening, eye/ear drops can be used.	26.9%	29.9%	23.7%	16.9%	2.7%
I should avoid taking over-the-counter medications when I am sick.	6.4%	21.1%	35.9%	27.3%	9.4%
Although over-the-counter medications are safe, you should consult a doctor before using them.	2.7%	5.8%	20.6%	53.7%	17.3%

### SM practice patterns

**Fig 2:**
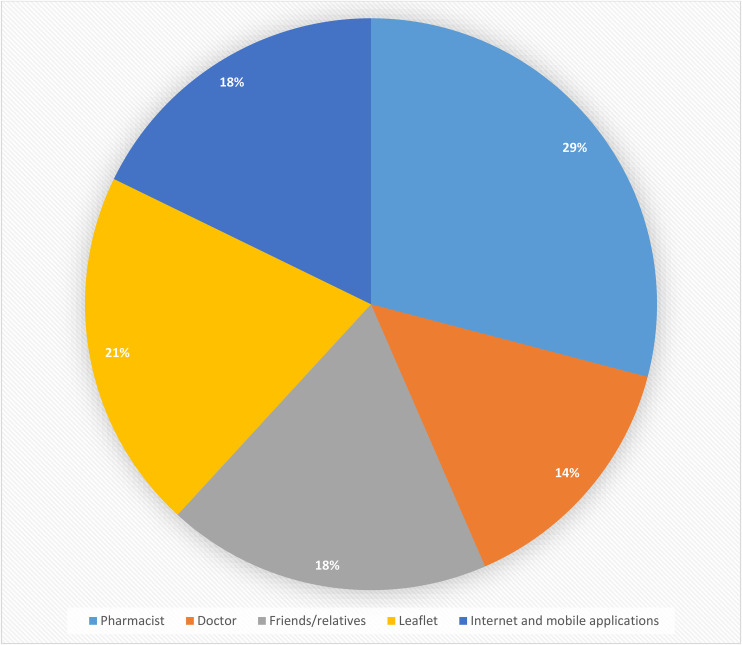
Sources of information before practicing SM with OTC drugs.

**Fig 3:**
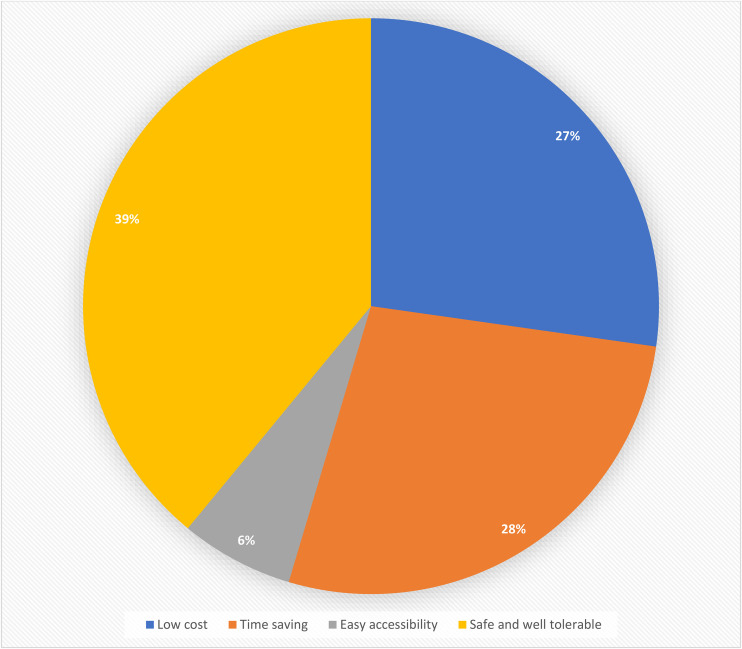
Reasons for practicing SM with OTC drugs.

**Fig 4:**
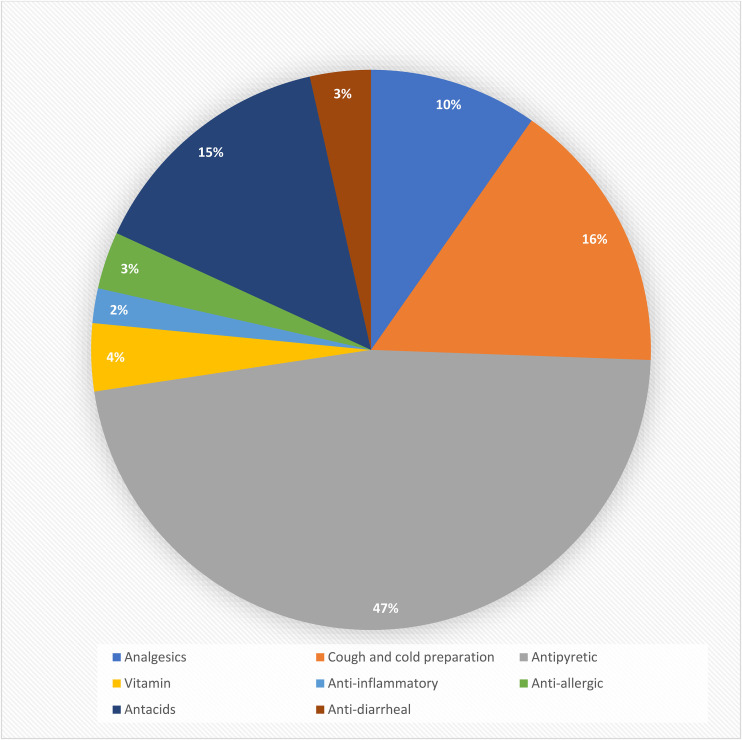
Medication categories preferred for SM practice.

Table 5 presents the patterns of SM practices among university students. More than half of participants (51.2%) consulted pharmacists before using OTC medications; see Fig 2. Most (65.7%) admitted to using OTC medications when symptoms were minor or manageable. Their reasons for opting for SM with OTC medications included perceived safety and tolerability, followed by low costs and time-saving; see Fig 3. Antipyretics were the preferred OTC medications among 46.9% of respondents; see Fig 4. Nearly one-third (29.7%) of the respondents had taken OTC medications in higher doses than recommended, while 23.7% reported adverse drug reactions.

**Table 5:** SM patterns.

Variable	Frequency	Percentage
**Who did you consult before using over-the-counter drugs? (n = 575) (multiple answer question)**		
Pharmacist	294	51.2%
Doctor	145	25.3%
Friends/relatives	186	32.3%
Leaflet	206	35.9%
Internet and mobile applications	180	31.3%
**When do you consume over-the-counter drugs? (n = 575)**		
When symptoms are minor/manageable (symptoms that do not disturb daily life and can be managed without medical intervention)	378	65.7%
Whenever I feel sick	117	20.4%
When I cannot visit a doctor	79	13.8%
**Common reason(s) for using over-the-counter drugs: (n = 575) (multiple answers question)**		
Low cost	251	43.7%
Time-saving	252	43.9%
Easy accessibility	59	10.2%
Safe and well-tolerable	360	62.6%
**Which categories of medications do you prefer for self-medication? (n = 575)**		
Analgesics	56	9.7%
Cough and cold preparation	91	15.8%
Antipyretic	270	46.9%
Vitamin	22	3.9%
Anti-inflammatory	12	2.0%
Anti-allergic	19	3.3%
Antacids	84	14.6%
Anti-diarrheal	20	3.5%
**Have you ever taken more than the recommended dose of an over-the-counter drug? (Yes) (n = 575)**	171	29.7%
**Have you ever experienced adverse effects from over-the-counter drugs? (Yes) (n = 575)**	136	23.7%

### Factors and reasons influencing SM

A Chi-squared test revealed no significant association between independent variables and SM practices; see Table 6. Moreover, the estimated odds ratio with a 95% CI did not show a statistically significant association across the study variables (p-value>0.05); see Supplementary Material S1 Table.

**Table 6:** Factors associated with SM.

Variable	Practicing SM (Frequency (%)) (n = 575)	Not Practicing SM (Frequency (%)) (n = 694)	P-value
**Gender**			
Male	262 (45.6%)	242 (34.9%)	0.843
Female	313 (54.4%)	452 (65.1%)	
**Year of study**			
First year	230 (40.0%)	296 (42.7%)	0.275
Second year	163 (28.3%)	197 (28.4%)	
Third year	85 (14.8%)	101 (14.6%)	
Fourth year	50 (8.7%)	64 (9.2%)	
Fifth year	24 (4.2%)	12 (1.7%)	
Sixth year	9 (1.6%)	9 (1.3%)	
Postgraduate	14 (2.4%)	15 (2.2%)	
**Monthly income category**			
Less than JOD 500	267 (46.4%)	323 (46.5%)	0.964
JOD 500–1000	191 (33.2%)	231 (33.3%)	
JOD 1001–1500	64 (11.1%)	72 (10.4%)	
JOD 1501 or more	53 (9.2%)	68 (9.8%)	
**Field of study**			
Medical field (medicine and surgery, dentistry, pharmacy or Doctor of Pharmacy, nursing, allied health sciences, and clinical nutrition and dietetics)	233 (40.5%)	301 (43.4%)	0.332
Non-medical field (engineering, computer sciences and information technology, business administration, arts and humanities, sciences, law, agriculture, tourism, and sport sciences)	342 (59.5%)	393 (56.6%)	
**Employment status**			
Unemployed	510 (88.7%)	636 (91.6%)	0.086
Employed	65 (11.3%)	58 (8.4%)	
**Do you have any history of chronic disease?**			
Yes	55 (9.6%)	56 (8.1%)	0.370
No	520 (90.4%)	638 (91.9%)	

Binary logistic regression analysis identified that fifth-year students were more likely than others to practice SM (p-value = 0.021). Similarly, a multiple logistic regression analysis identified that fifth-year students were more likely to practice SM (p-value = 0.011). Additionally, better knowledge was associated with higher odds of practicing SM (aOR = 1.86, 95% CI: 1.47–2.35, p = 0.0001), and participants with a positive attitude were more likely to practice SM (aOR = 1.51, 95% CI: 1.20–1.89, p < 0.001); see Table 7. This indicates an independent association when adjusting for other variables.

**Table 7:** Predictors of SM practice among university students.

Variable	Odds ratio of SM practice	95% confidence interval	Adjusted odds ratio of SM practice	95% confidence interval	
**Gender**					
Female (Reference category)	1.00		1.00		
Male	0.95 (0.65-1.41)	0.812	0.87 (0.57-1.33)	0.533	
**Year of study**					
First year	1.00		1.00		
Second year	1.07 (0.81-1.40)	0.648	0.99 (0.75-1.31)	0.985	
Third year	1.08 (0.77-1.52)	0.642	1.05 (0.74-1.48)	0.7745	
Fourth year	1.01 (0.67-1.51)	0.979	0.95 (0.63-1.45)	0.844	
Fifth year	2.57 (1.26-5.26)	0.009**	2.38 (1.14-4.95)	0.021*	
Sixth year	1.29 (0.50-3.29)	0.599	1.13 (0.42-3.02)	0.799	
Postgraduate	1.20 (0.57-2.54)	0.631	1.04 (0.47-2.30)	0.908	
**Field of study**					
Medical field (Reference category)	1.00		1.00		
Non-medical field	1.12 (0.90-1.41)	0.306	1.19(0.94-1.51)	0.135	
**Employment status**					
Unemployed (Reference category)	1.00		1.00		
Employed	1.40 (0.96-2.03)	0.078	1.41 (0.94-2.12)	0.095	
**Monthly income for the family**					
Less than JOD 500 (Reference category)	1.00		1.00		
JOD 500–1000	1.00 (0.78-1.29)	0.998	0.98 (0.75-1.26)	0.877	
JOD 1001–1500	1.08 (0.74-1.56)	0.703	0.98(0.66-1.45)	0.943	
JOD 1501 or more	0.94 (0.64-1.40)	0.770	0.87 (0.58-1.31)	0.521	
**Chronic disease history**					
No (Reference category)	1.00		1.00		
Yes	1.21 (0.82-1.78)	0.348	1.09 (0.73-1.64)	0.645	
**Attitude towards SM practices**					
Negative attitude, defined as an attitude score below 29.6 (Reference category)	1.00		1.00		
Positive attitude	1.47 (1.18-1.84)	0.0001***	1.51 (1.20-1.89)	0.0001***	
**Knowledge of SM practices**					
Poor knowledge (Reference category)	1.00		1.00		
Good knowledge	1.82 (1.44-2.29)	0.0001**	1.86 (1.47-2.35)	0.0001***	

*p < 0.05, **p < 0.01, ***p < 0.001.

## Discussion

This study aimed to investigate the prevalence of SM practices among university students in Jordan. The findings of this study indicate that 45.3% of these university students engage in SM practices, a rate comparable to studies conducted in Bahrain (44.8%), Tabuk city, KSA (43.2%), and New Delhi, India (44.5%). [3,16,17]. This rate is lower than that in studies from other countries, including Syria (67.3%), Lebanon (79.1%), and Palestine (87.7%) [18,19]. However, the rate of using SM in this study was higher than that found in Brazil (16.1%) [20]. These differences may be attributed to cultural variations, as students may use SM practices in countries with limited access to healthcare services. Moreover, in some countries, stricter rules and regulations prevent the purchase of medications without a prescription. This may contribute to a lower prevalence rate of SM practices compared to countries that have no such strict rules [21].

In this study, fifth-year students had a higher probability of practicing SM than other students (aOR = 2.38, 95% CI: 1.14–4.95, p = 0.021). This could be because they are more confident, having more knowledge about various aspects of science than more junior students. This might increase the likelihood of them practicing SM. Meanwhile, the Internet has become one of the main sources of medical information and a guide to SM practices among different populations [10]. Another possible cause of this higher probability is that fifth-year students are approaching graduation and often experience higher levels of academic stress due to exams. This pressure could lead to practicing SM to handle minor ailments such as headaches and mild anxiety.

Surprisingly, in this study, other sociodemographic factors such as gender, field of study, and monthly family income did not show a statistically significant influence on SM practices among university students (p > 0.05). This differed from previous studies, in which more female students practiced SM than males [22,23]. A previous study in Saudi Arabia found that a higher income level was associated with a greater tendency toward SM, in terms of the impact of the monthly income level [22]. Students tend to practice SM as a cost-saving strategy due to their limited financial resources and to avoid expenses associated with doctor visits. This SM behavior may reflect economic barriers to accessing formal healthcare. A previous study from Saudi Arabia demonstrated that medical students practice SM more than non-medical students. This was explained by the students’ greater access to knowledge about health conditions, medications, and their adverse effects, which led them to be more confident in practicing this behavior [23].

In this study, the majority of the participating students (83.5%) were cautious about OTC medications, recognizing the risks associated with combining OTC and prescribed drugs. This study also found that 95.5% believed that OTC medications should not be used after the expiration date. These findings align with the findings of previous studies conducted in Nigeria and KSA [2,23]. Expired medications undergo chemical changes that alter their chemical structures. This can lead to a reduction in their potency and safety. Using expired medications can cause health risks or undesirable side effects and fail to yield the intended therapeutic benefits [24].

Regarding why people self-medicate, this study found that perceived safety and tolerability, saving time and low cost were the primary factors contributing to the practice. Earlier studies had similar findings [19,25]. Further, the present study found that antipyretics were the most common SM drugs (46.9%). This finding was similar to a previous study [26] in which these medications were followed by cough and cold preparations (15.8%) [22,26]. The frequent use of antipyretics among university students may be caused by their common need to manage stress-induced ailments, including tension headaches and other types of pain. These are frequent health issues among this population, particularly during exams. However, this finding differed from that of a Syrian study, which found that using vitamins was the most common SM practice [27]. This variation might be due to different regional socioeconomic cultures.

This study found that self-medicating with antibiotics (29.4%) was the least common practice. This could be due to a high level of awareness concerning antibiotic resistance [28,29]. A further finding was that 93% of the respondents were aware of the caution that needs to be exercised in pregnancy. This was different from the findings of a previous study in Jordan, which reported that pharmacy students had low levels of knowledge regarding medication use in pregnancy [30]. Moreover, the present study found that 51.2% of university students consulted pharmacists before using OTC medications. Most participants (65.7%) reported that they used OTC drugs for minor or manageable symptoms. This finding relates to the vital roles of pharmacists in practicing patient counselling.

This study found that better knowledge was associated with higher odds of practicing SM (aOR = 1.86, 95% CI: 1.47–2.35, p = 0.0001), and participants with a positive attitude were more likely to practice SM (aOR = 1.51, 95% CI: 1.20–1.89, p < 0.001). These findings suggest that having a higher level of knowledge about the proper use of medications and their associated potential risks increases participants’ confidence and the likelihood of practicing SM. Having a positive attitude towards SM also increases the probability of such practices. This supports the finding that having good knowledge of SM and a positive attitude towards it significantly shapes individuals’ medication use behavior. These findings aligned with previous literature on the association between individuals’ SM knowledge, attitude, and practices [31]. A previous study in Spain demonstrated that individuals who have a good perception of the efficacy of SM were more likely to practice SM [32].

SM is becoming more important in the healthcare sector. It encourages patient autonomy in terms of their healthcare-related decision-making, specifically, regarding the management of minor ailments. It also offers benefits to healthcare systems by reducing the costs of prescribed drugs and healthcare professionals’ visits, increasing access to medication, and facilitating a more effective use of clinical skills and healthcare resources. Nevertheless, SM is linked to disadvantages, including drug interactions, polypharmacy, a potential for excessive drug dosage, and misdiagnosis (inappropriate self-diagnosis). The provision of healthcare education and information about the use of SM is proposed to minimize the risks and maximize the benefits of this practice. This could be achieved through a partnership among patients, physicians, and pharmacists.

This study has limitations. The causal relationship across the study meant variables could not be examined due to the cross-sectional study design. The use of convenience sampling means selection bias may have occurred, as some of the target population might have been missed due to the use of an online survey (e.g., students who do not use social media platforms). The use of such surveys is not free from bias and may influence the generalizability of the study findings. In this case, specific sub-groups may have been underrepresented, such as students in their last year of study. For example, fifth-year students comprised only 2.8% of the study sample. This warrants caution when interpreting the study findings related to this group of students. Furthermore, this research may have been influenced by social durability bias, as some participants might have answered the questions in a socially appealing manner. Additionally, this research did not examine certain demographic characteristics (such as age, marital status, family members working in the healthcare system, and student accommodation with families or in hostels) that play a significant role in attitude, awareness, and the practice of SM. Therefore, the study findings should be interpreted with care. Furthermore, the regression model included demographic characteristics concerning knowledge and attitude. Important behavioral and contextual determinants of SM, such as health literacy, were not included. Finally, limitations in the psychometric evaluation of the survey items should be noted. Two of the extracted factors consist of single items, preventing a reliability assessment using Cronbach’s alpha. Moreover, the internal consistency for the first two factors was modest (alpha = 0.4 and 0.57), which limits the interpretability and stability of the factor structure. This research recommends that future studies investigate the impact of students’ socioeconomic characteristics and their influence on SM practices. Future research should also examine a wider range of participants’ socioeconomic characteristics, such as health insurance status, age, education, occupation, marital status, number of children at home, distance to the nearest hospital and pharmacy, and information about family members employed in the health sector. In addition, SM patterns, including the frequency and outcome of SM, the prevalent illnesses for which SM is practiced, and the type of drugs used, should be examined [33].

## Conclusion

This study provides descriptive insights into the demographic factors associated with SM practices among university students. While fifth-year students showed higher odds of engaging in SM, these associations should be interpreted cautiously due to the cross-sectional study design and the limited set of predictors assessed. The findings suggest areas where universities could strengthen health education efforts, including raising awareness about the safe use of medication, appropriate dosages, and risks of drug interactions. Future studies should incorporate broader behavioral and contextual determinants to understand the predictors of SM.

## Supporting information

S1 Table Risk estimation for the study variables. (DOCX)
