# Supplementary material for: Prevalence, knowledge, attitudes, and practices concerning self-medication with over-the-counter drugs among university students in Jordan: A cross-sectional study
Source: PLoS One. 2026 Jan 28;21(1):e0339915. doi: 10.1371/journal.pone.0339915 (PMC12851458; doi:10.1371/journal.pone.0339915)
Supplement: S1 Table — (DOCX) [file pone.0339915.s001.docx]

**Supplementary material:**

Table S1: Risk estimation for the study variables

| Variables | Estimated risk  (Odds ratio) | Confidence interval | |
| --- | --- | --- | --- |
|  |  | Lower limit | Upper limit |
| **Gender** | | | |
| Females | 0.954 | 0.647 | 1.407 |
| **Year of study** | | | |
| First to third year | 1.205 | 0.889 | 1.634 |
| **Field of study** | | | |
| Medical field | 1.124 | 0.898 | 1.407 |
| **Employment status** | | | |
| Unemployed | 1.398 | 0.963 | 2.029 |
| **Monthly income for the family** | | | |
| Less than 1000 JD | 1.011 | 0.768 | 1.331 |
| **Chronic disease history:** | | | |
| No | 1.205 | 0.816 | 1.779 |
